# Cell death serum biomarkers are early predictors for survival in severe septic patients with hepatic dysfunction

**DOI:** 10.1186/cc7923

**Published:** 2009-06-18

**Authors:** Stefan Hofer, Thorsten Brenner, Christian Bopp, Jochen Steppan, Christoph Lichtenstern, Jürgen Weitz, Thomas Bruckner, Eike Martin, Ursula Hoffmann, Markus A Weigand

**Affiliations:** 1 Department of Anaesthesiology, University of Heidelberg, Im Neuenheimer Feld 110, D-69120 Heidelberg, Germany; 2 Department of Anaesthesiology and Intensive Care Medicine, University of Gießen, Rudolf-Buchheim-Strasse 7, D-35392 Gießen, Germany; 3 Department of Surgery, University of Heidelberg, Im Neuenheimer Feld 110, D-69120 Heidelberg, Germany; 4 Institute of Medical Biometry and Informatics, University of Heidelberg, Im Neuenheimer Feld 305, D-69120 Heidelberg, Germany; 5 First Medical Department, Medical Faculty Mannheim, University of Heidelberg, Theodor-Kutzer-Ufer 1-3, D-68167 Mannheim, Germany

## Abstract

**Introduction:**

Severe sepsis, septic shock, and resulting organ failure represent the most common cause of death in intensive care medicine, with mortality ranging from 40% to 70%. It is still unclear whether necrosis or apoptosis plays the predominant role in severe sepsis. Determining the prevalent mode of cell death would be valuable, as new therapeutic agents (eg, antiapoptotic drugs such as caspase inhibitors) may improve unsatisfactory outcomes in patients with severe sepsis. Furthermore, the prognostic value of newly developed cell death serum biomarkers is of great interest.

**Methods:**

In total, 147 patients (101 patients with severe sepsis, 28 postoperative patients after major abdominal surgery, 18 healthy volunteers) were enrolled. Baseline and clinical data were evaluated. Blood samples from patients with severe sepsis were collected at the time of sepsis diagnosis, and 48 and 120 hours later; samples from healthy volunteers were collected once, and from postoperative patients, once immediately after surgery. We measured caspase-cleaved and uncleaved cytokeratin-18 (CK-18, intermediate filament protein) as a marker of cell death, isolated CK-18 fragments as a marker of apoptosis, as well as IL-6, soluble vascular cell adhesion molecule, and soluble intercellular adhesion molecule.

**Results:**

Age and sex of patients with severe sepsis and postoperative patients were comparable, whereas healthy volunteers were significantly younger. In healthy volunteers, the mode of cellular turnover was primarily apoptotic cell death. Postoperative patients showed comparable levels of apoptotic activity, but necrotic cell death was markedly increased, probably due to surgical tissue injury. In contrast, patients with severe sepsis, and especially non-survivors of the septic group showed increased levels of markers for both apoptotic and necrotic cell death. In severe septic patients with liver dysfunction, necrosis is increased relative to severe septic patients with intact hepatic function. For severe septic patients with liver dysfunction, a cut-off value for caspase-cleaved and uncleaved cytokeratin-18 could be calculated, in order to identify patients at high risk for death due to severe sepsis.

**Conclusions:**

The measurement of caspase-cleaved and uncleaved cytokeratin-18 appears to be an early predictor for survival in severe septic patients with hepatic dysfunction. Furthermore, the loss of parenchymal cells due to necrosis may be the primary mode of cell death in these patients. This may limit possible therapeutic options.

## Introduction

Severe sepsis, septic shock, and the resulting multiple organ failure/dysfunction syndrome represent an ongoing challenge in intensive care units [1-5]. With mortality ranging from 40% to 70%, septic shock is the most common cause of death in intensive care medicine [2,6].

The pathogenesis of multiple organ failure/dysfunction syndrome in patients with severe sepsis is a multifactorial process. Global tissue hypoxia due to an imbalance between systemic oxygen delivery and peripheral oxygen demand plays an important role. The resulting dysfunction and death of epithelial cells is detrimental to patients' survival in sepsis [7-13]. There is increasing evidence that, in addition to cellular necrosis, the apoptotic mode of cell death in critically ill patients plays a pivotal role in the pathogenesis of sepsis syndrome [14]. The key mediators of apoptosis are caspases, leading to the caspase-dependent pathway of apoptotic cell death. Caspases are intracellular cysteine proteases that cleave various substrates including structural proteins such as cytokeratins [15]. In addition to caspase-dependent cellular apoptosis, a caspase-independent pathway exists [16-20]. Despite the absence of caspase-specific proteolytic activity, the dying cells retain the main cytoplasmic features of classic caspase-dependent apoptosis (ie, cell shrinkage, membrane blebbing, phosphatidylserine externalization, and dissipation of the mitochondrial inner transmembrane potential). Furthermore, overlapping forms of apoptotic and necrotic modes of cell death have been reported [21].

Cytokeratin 18 (CK-18) is a structural protein of the intermediate filament group present in most simple epithelial and parenchymal cells [22,23]. Induction of caspase-dependent apoptosis leads to cleavage of CK-18 at various sites by caspases 3, 6, 7, and 9 [24]. The resulting fragments of CK-18 are released into the plasma after plasma membrane disintegration at later stages of apoptosis [25,26]. Fragments of CK-18 are more specific for apoptotic cell death; in contrast, during necrosis, only full-length CK-18 is released into the plasma. Determination of the predominant mode of cell death is facilitated by using a recently developed monoclonal antibody (M30) that recognizes caspase-cleaved CK-18 fragments containing the CK-18 Asp 396 neoepitope without detecting native or intact CK-18 [24,27] for assessing apoptosis, in combination with measuring total CK-18 as an indirect marker for necrosis [28].

The aim of this study was to measure serum concentrations of CK-18 neoepitope in relation to total CK-18, to detect the leading mode of cell death in patients with severe sepsis, postoperative patients after major abdominal surgery, and healthy volunteers.

## Materials and methods

The observational clinical study was approved by the local ethics committee and was conducted in the surgical intensive care units of the university hospitals of Heidelberg and Mannheim, Germany. All study and control patients or their legal designees gave written informed consent. In total, 147 patients in three groups were enrolled in the study. The three groups included 101 patients with severe sepsis (the septic group), 28 patients after major abdominal surgery (the postoperative group), and 18 healthy volunteers (the volunteer group; Table 1). The 101 patients were classified as having severe sepsis based on the criteria of the International Sepsis Definitions Conference [29]. Patients were eligible for enrollment with an onset of sepsis syndrome of 24 hours or less. The initial blood draw was also performed within this period. In contrast, patients with an onset of sepsis syndrome of more than 24 hours were excluded from the study.

**Table 1:** Baseline data of 101 patients in the septic group, 28 patients in the postoperative group and 18 individuals in the volunteer group

Septic group	
Demographic data	
Age, years	65.9 ± 12.4 (range = 28 to 97; median = 68; interquartile range = 60 to 74)
Male sex	60 (59.4%)
Primary site of infection/septic focus	
Lung	59 (58.4%)
Gastrointestinal tract	4 (4.0%)
Genitourinary tract	11 (10.9%)
Surgical site	7 (6.9%)
Other	11 (10.9%)
Unknown	9 (8.9%)
Outcome	
Survivor	52 (51.5%)

Postoperative group	

Demographic data	
Age, years	62.3 ± 14.2 (range = 37 to 84; median = 64; interquartile range = 51.5 to 73)
Male sex	15 (53.6%)
Primary site of surgery	
Pancreas	13 (46.4%)
Colon	5 (17.9%)
Liver	2 (7.1%)
Genitourinary	3 (10.7%)
Other abdominal	5 (17.9%)

Volunteer group	

Demographic data	
Age, years	34.5 ± 8.6
Male sex	10 (55.6%)

Data are presented by number (%) except for age, which is presented by mean ± standard deviation.

The management of patients with severe sepsis in the intensive care unit included early goal-directed therapy (according to Rivers and colleagues [30]), elimination of the septic focus, and administration of broad-spectrum antibiotics [31,32]. Patients with central nervous system disorders (eg, traumatic brain injury due to severe trauma, as indicated by a Glasgow Coma Scale ≤14, according to Sequential Organ Failure Assessment (SOFA) score), renal disorders (as indicated by a serum-creatinine ≥1.2 mg/dl or 20.5 μmol/L, according to SOFA score) as well as liver diseases (as indicated by a serum-bilirubin ≥1.2 mg/dl or 20.5 μmol/L, according to SOFA score) prior to the onset of sepsis were excluded from the study. The second group included 28 patients undergoing major abdominal surgery, with negative parameters for systemic inflammatory response syndrome (Table 1). As a control group, we chose 18 healthy young volunteers with no signs of infection (Table 1). After enrollment of patients, data were masked as to group status to avoid potential bias.

Blood samples from patients with severe sepsis were collected after the diagnosis of sepsis, and 48 and 120 hours later. Relevant baseline data (demographic data, primary site of infection, outcome) and clinical data (systolic, diastolic, and mean arterial pressure, central venous pressure, heart rate, administration of norepinephrine, corticosteroids, fraction of inspired oxygen, Horowitz index (oxygenation ratio), temperature) were collected. In the septic group, severity of illness was estimated using the Acute Physiology and Chronic Health Evaluation (APACHE) II score. Patients with sepsis were reevaluated for survival 90 days after enrollment in the study. This evaluation was performed using available hospital records. In the case of the patient's discharge from hospital, the family doctor was contacted. If necessary, we made contact with the patient himself. Blood samples from the postoperative group were collected once immediately after surgery, and from the volunteer group, once.

After blood collection, serum of all study participants was immediately obtained by centrifugation, transferred into cryotubes, and stored at -80°C until further processing. Serum tests for creatinine, urea, bilirubin, pH, arterial oxygen partial pressure, base excess, lactate, leukocytes, and C-reactive protein were performed at the same time.

Measurement of cleaved and uncleaved soluble CK-18 (M65 antigen), also known as total CK-18, represented overall cell death due to both apoptosis and necrosis. For the quantitative determination of total CK-18 in serum, we used the M65 ELISA kit (Peviva AB, Bromma, Sweden) according to the manufacturer's instructions. The M65 ELISA uses two monoclonal antibodies (clones M5 and M6) specific for conventional epitopes on CK-18, present on both intact/uncleaved and cleaved CK-18. Serum samples react with solid phase-catcher M6 antibody directed against CK-18 and horseradish peroxidase-conjugated M5 antibody directed against a different epitope on CK-18.

Measurement of the caspase-generated neoepitope of CK-18 (M30 antigen) represented cell death due specifically to apoptosis. For the quantitative determination of the caspase-generated neoepitope of CK-18, we used the M30-Apoptosense ELISA kit (Peviva AB, Bromma, Sweden) according to the manufacturer's instructions. This ELISA uses a monoclonal antibody that recognizes an epitope on the 238–396 fragment of CK-18 as catcher and a horseradish peroxidase-conjugated M30 as detector. Serum concentrations of the antigens in each sample were calculated from the accompanying calibration curves [24]. Samples with high values outside the standard curve were diluted, yielding satisfying linearity.

Furthermore, different serum biomarkers were measured in order to determine the ongoing inflammatory response (IL-6) and cellular activation (soluble vascular cell adhesion molecule-1 (sVCAM-1), soluble intercellular adhesion molecule-1 (sICAM-1)) in sepsis syndrome [33,34]. We used ELISA kits to determine serum concentrations of Il-6 (R&D Systems, Minneapolis, MN, USA), sVCAM-1 (Bender MedSystems, Vienna, Austria), and sICAM-1 (Bender MedSystems, Vienna, Austria).

All assays were performed in duplicate. The resulting study data were entered into an electronic database (Microsoft^® ^Excel 2002, Unterschleißheim, Germany) and evaluated using SPSS software (Statistical Product and Services Solutions, version 16.0, SPSS Inc, Chicago, IL, USA).

Categorical data were summarized by means of absolute and relative frequencies (counts and percentages). Quantitative data were summarized using the number of observations, mean and standard deviation, minimum, median with quartiles, or differences of the quartiles and maximum.

Wherever appropriate, data were visualized using box-and-whisker plots. The Kolmogorov-Smirnov test was applied to check for normal distribution. Due to non-normally distributed data, non-parametric methods for evaluation were used (chi-squared test for categorical data, Mann-Whitney U test for continuous data). Logistic regression analysis was performed with suitable parameters to determine the prognostic value of each parameter with regard to survival. Furthermore, a receiver operating characteristic (ROC) curve was established with suitable parameters, in order to create cut-off values to determine the prognostic value of each parameter with regard to survival. Correlation analysis was performed calculating Pearson's correlation coefficient. A *P *< 0.05 was considered statistically significant. Concerning symbolism and higher orders of significance: * *P *< 0.05: ** *P *< 0.01: *** *P *< 0.001.

## Results

Age and sex of patients in the septic and postoperative groups were comparable (Table 1). In the septic group, patients who survived or died showed no significant differences concerning their demographic data (data not shown). In contrast, healthy volunteers were significantly younger compared with the septic and postoperative groups (Table 1).

The primary site of infection in the septic group was the respiratory tract (59 patients, 58.4%), followed by the genitourinary tract (11 patients, 10.9%), surgical site (7 patients, 6.9%), and gastrointestinal tract (4 patients, 4.0%). In nine patients with sepsis, the septic focus remained unknown (Table 1). A positive culture from the site of infection was obtained in 73% of all septic patients. In these patients, cultures were found to be Gram-negative in 53% and Gram-positive in 47%. Patients in the postoperative group primarily underwent surgery of the pancreas (46.4%), whereas surgery of the colon (17.9%), liver (7.1%), and the genitourinary tract (10.7%) were less frequent (Table 1). In the septic group, 52 of 101 patients (51.5%) survived (Table 1). No one in the postoperative or volunteer groups died during the study.

Levels of IL-6 were significantly elevated in the septic and postoperative groups compared with the volunteer group (Table 2). Furthermore, IL-6 was the only inflammatory marker that had significantly different levels between the postoperative and volunteer groups. In the septic group, the level of IL-6 decreased significantly by 120 hours, but still remained significantly higher than the volunteer group (data not shown). The level of sICAM-1 was significantly elevated at the time of diagnosis of sepsis compared with levels in the postoperative and volunteer groups (Table 2), and it remained significantly elevated at 48 and 120 hours. However, no significant changes in sICAM-1 levels occurred within the first 120 hours in the septic group (data not shown). Levels of sVCAM-1 did not differ in the three groups (Table 2).

**Table 2:** Comparison of inflammatory marker levels and cytokeratin measurements in the volunteer, postoperative, and septic groups at baseline

	Healthy (n = 18)	Postoperative (n = 28)	Sepsis (n = 101)
IL-6 (pg/ml)	0.0; 0.0 to 0.8	216.7; 48.8 to 360.5	160.5; 58.8 to 448.8
*P *values	Healthy vs. Postoperative: *P *< 0.001***		
	Healthy vs. Sepsis: *P *< 0.001***		
	Postoperative vs. Sepsis: *P *= 0.604		
sICAM-1 (ng/ml)	219.6; 195.2 to 285.1	213.7; 192.3 to 293.8	444.7; 330.3 to 665.5
*P *value	Healthy vs. Postoperative: *P *= 0.819		
	Healthy vs. Sepsis: *P *< 0.001***		
	Postoperative vs. Sepsis: *P *< 0.001***		
sVCAM-1 (ng/ml)	1524.7; 991.2 to 2038.0	1268.0; 1167.7 to 1550.8	1147.9; 883.5 to 2047.4
*P *value	Healthy vs. Postoperative: *P *= 0.545		
	Healthy vs. Sepsis: *P *= 0.280		
	Postoperative vs. Sepsis: *P *= 0.450		
Total CK-18 (U/l)	241.9; 216.9 to 285.3	558.7; 465.6 to 793.0	1643.8; 1096.5 to 2633.5
*P *value	Healthy vs. Postoperative: *P *< 0.001***		
	Healthy vs. Sepsis: *P *< 0.001***		
	Postoperative vs. Sepsis: *P *< 0.001***		
CK-18 fragments (U/l)	143.7; 134.4 to 168.1	116.0; 106.6 to 165.1	392.6; 258.4 to 654.5
*P *value	Healthy vs. Postoperative: *P *= 0.250		
	Healthy vs. Sepsis: *P *< 0.001***		
	Postoperative vs. Sepsis: *P *< 0.001***		
Ratio	0.58; 0.55 to 0.67	0.22; 0.18 to 0.25	0.24; 0.14 to 0.35
*P *value	Healthy vs. Postoperative: *P *< 0.001***		
	Healthy vs. Sepsis: *P *< 0.001***		
	Postoperative vs. Sepsis: *P *= 0.507		

Data are presented by median and interquartile range (Q1 to Q3).

CK = cytokeratin; sICAM = soluble intercellular adhesion molecule-1; sVCAM = soluble vascular cell adhesion molecule-1.

*** *P *< 0.001.

**Figure 1:**
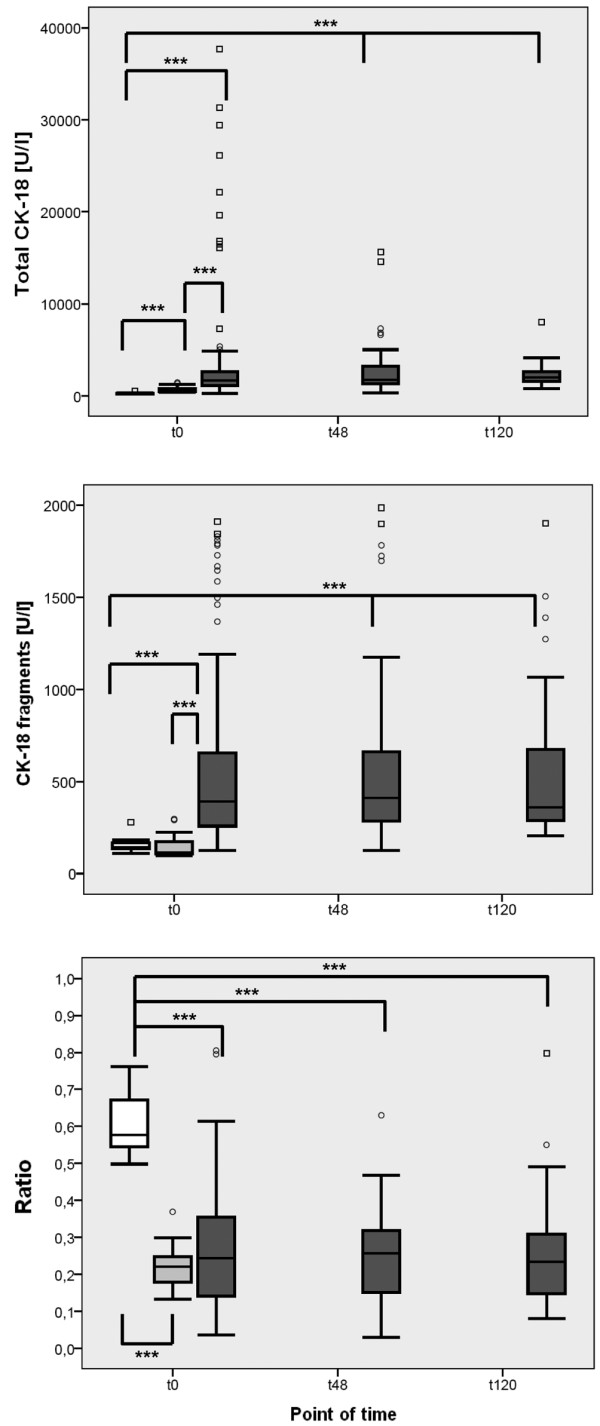
Comparison of cytokeratin measurements in the volunteer, postoperative, and septic groups at baseline and at 48 and 120 hours in the septic group. Concentrations were measured of total cytokeratin-18 (CK-18) and CK-18 fragments (CK-18-F), and the ratio of CK-18-F to total CK-18 was calculated from the sera of healthy volunteers ('Healthy', n = 18, white box), postoperative patients after major abdominal surgery ('Post-op', n = 28, light grey box), and patients with sepsis ('Sepsis', n = 101, dark grey box), at t0 (measured once for the volunteer group, immediately after surgery for the postoperative group, and at the time of diagnosis of sepsis for the sepsis group). In addition, for the septic group, the two other times of data collection are represented, t48 and t120 for 48 and 120 hours, respectively, after the diagnosis of sepsis. Data in box plots are given as median, 25^th ^percentile, 75^th ^percentile, and the 1.5 interquartile range. Outliers are shown in form of circles (1.5 to 3 interquartile ranges above 75^th ^percentile or below 25^th ^percentile) or rectangles (>3 interquartile ranges above 75^th ^percentile or below 25^th ^percentile). *** *P *< 0.001.

Levels of total CK-18 and CK-18 fragments in the septic group at baseline were significantly higher than in the postoperative and volunteer groups (Figure 1 and Table 2). Levels of CK-18 fragments were comparable in the postoperative and volunteer groups, but levels of total CK-18 were significantly higher in the postoperative group (Figure 1 and Table 2). The ratio of CK-18 fragments and total CK-18 was significantly higher in the volunteer group compared with the septic and postoperative groups. In contrast, the ratio was not significantly different in the septic and postoperative groups (Figure 1 and Table 2). In the septic group, levels of CK-18 fragments and total CK-18 did not change significantly by 120 hours (Figure 1).

In comparing subgroups of patients in the septic group who did and did not survive, the APACHE II score was significantly higher at the time of diagnosis of sepsis in patients who ultimately died (median score, 30.5 in patients who died versus 26 in patients who survived; *P *= 0.003**). Logistic regression analysis revealed a significant association between the initial APACHE II score and survival. Concerning organ dysfunction, both septic subgroups showed comparable reduction of pulmonary function and comparable occurrence of acute renal failure. In this context, creatinine and urea were increased but did not differ significantly between the two subgroups. Bilirubin as a marker of liver function was increased but did not differ significantly between the two subgroups. Lactate was significantly higher in the non-surviving subgroup (*P *= 0.009**), whereas the rest of the parameters of acid-base metabolism were comparable. Both subgroups were comparable concerning hemodynamic parameters (mean arterial pressure, central venous pressure), but non-survivors showed an increased heart rate and received more vasoactive medication (norepinephrine) to maintain sufficient circulation. Both subgroups received comparable amounts of corticosteroids (data not shown).

Routine inflammatory markers (C-reactive protein, leukocytes) were not significantly different between the two subgroups. In contrast, maximum body temperature (*P *= 0.017*) was significantly higher in non-surviving patients (data not shown). Levels of sICAM-1, sVCAM-1, and IL-6 were comparable between the surviving and non-surviving subgroups at the time of diagnosis of sepsis, but in the non-surviving subgroup at later stages, levels were markedly (sICAM-1) or significantly (IL-6/sVCAM-1) higher at 48 hours and 120 hours (Table 3) in comparison to the surviving subgroup.

**Table 3:** Comparison of inflammatory marker levels and cytokeratin measurements in survivors and non-survivors in the septic group at baseline and at 48 and 120 hours

		Survivor (n = 52)	Non-survivor (n = 49)	*P *value
IL-6 (pg/ml)	t0	104.5; 34.0 to 430.0	249.4; 96.5 to 460.2	0.099
	t48	21.0; 8.0 to 46.0	82.1; 37.4 to 188.5	0.001**
	t120	17.2; 8.9 to 38.3	71.6; 15.7 to 107.1	0.01*
*P *value	t0-t48-t120	< 0.001***	0.005**	
sICAM-1 (ng/ml)	t0	447.5; 323.14 to 663.6	399.8; 327.9 to 655.4	0.841
	t48	434.1; 308.6 to 613.6	683.2; 348.3 to 1003.7	0.067
	t120	467.8; 320.4 to 593.0	630.2; 419.2 to 933.3	0.083
*P *value	t0-t48-t120	0.432	0.829	
sVCAM-1 (ng/ml)	t0	1146.3; 911.3 to 1975.7	1227.5; 857.2 to 2242.5	0.837
	t48	882.0; 569.6 to 1236.1	1275.1; 1040.0 to 2799.2	0.027*
	t120	748.5; 639.1 to 1202.6	1685.5; 958.7 to 2201.2	0.021*
*P *value	t0-t48-t120	0.097	0.505	
Total CK-18 (U/l)	t0	1581.9; 1030.0 to 2152.0	2006.5; 1169.9 to 4611.6	0.038*
	t48	1579.5; 1359.8 to 2058.7	2007.1; 1239.0 to 4488.8	0.212
	t120	1829.6; 1581.9 to 2076.9	2533.0; 1675.2 to 3535.5	0.073
*P *value	t0-t48-t120	0.396	0.814	
CK-18 fragments (U/l)	t0	357.7; 248.8 to 554.8	475.4; 301.5 to 1028.2	0.035*
	t48	355.4; 229.0 to 455.46	603.6; 410.7 to 973.5	0.007**
	t120	324.6; 283.7 to 499.9	508.5; 314.8 to 881.3	0.108
*P *value	t0-t48-t120	0.717	0.939	
Ratio	t0	0.3; 0.16 to 0.33	0.2; 0.12 to 0.36	0.436
	t48	0.2; 0.15 to 0.30	0.3; 0.15 to 0.35	0.651
	t120	0.2; 0.15 to 0.27	0.3; 0.15 to 0.36	0.534
*P *value	t0-t48-t120	0.405	0.939	

Data are presented by median and interquartile range (Q1 to Q3).

CK = cytokeratin; sICAM = soluble intercellular adhesion molecule-1; sVCAM = soluble vascular cell adhesion molecule-1.

* *P *< 0.05; ** *P *< 0.01; *** *P *< 0.001.

At the time of diagnosis of sepsis, levels of total CK-18 and CK-18 fragments were significantly higher in the non-surviving subgroup. The levels of CK-18 fragments remained significantly higher at 48 hours and decreased to comparable values at 120 hours. At 120 hours, levels of total CK-18 in non-surviving patients were still higher but not statistically significantly (*P *= 0.073). The ratio of CK-18 fragments to total CK-18 was comparable between the two subgroups at each time (Table 3).

**Figure 2:**
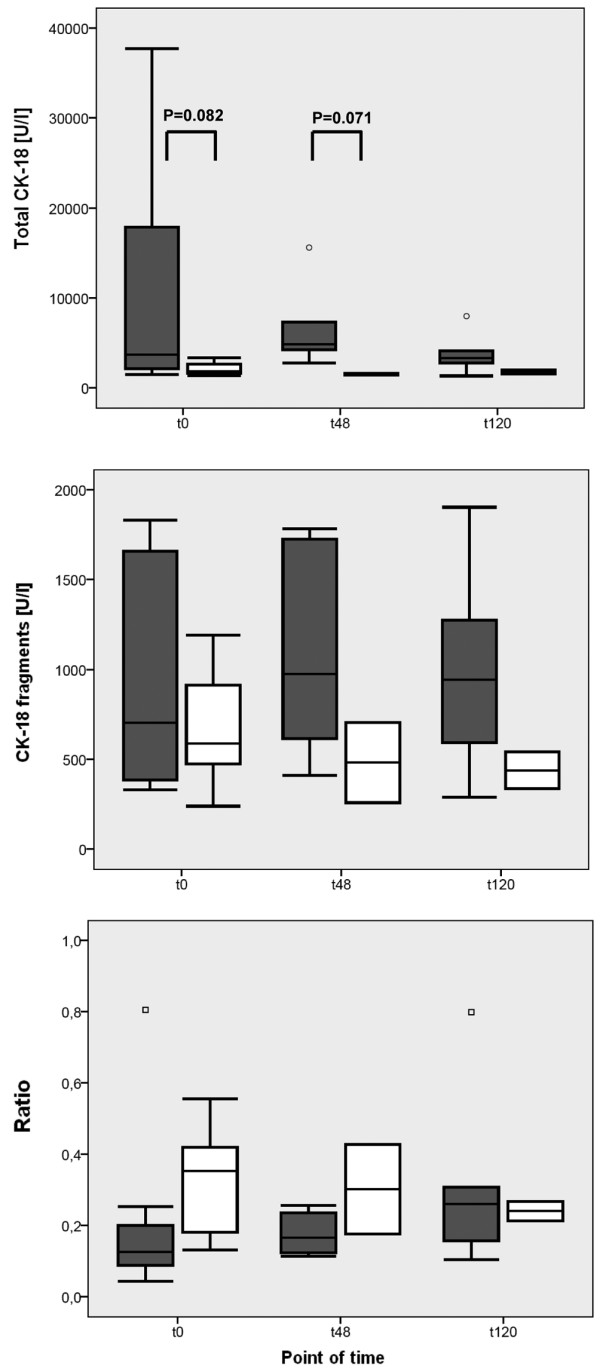
Comparison of cytokeratin measurements in survivors and non-survivors with impaired liver function in the septic group at baseline and at 48 and 120 hours. Concentrations were measured of total cytokeratin-18 (CK-18) and CK-18 fragments (CK-18-F), and the ratio of CK-18-F to total CK-18 was calculated from the sera of survivors (white box) and non-survivors (dark grey box) of the septic group with impaired liver function (bilirubin ≥1.2 mg/dL or 20.5 μmol/L according to Sequential Organ Failure Assessment (SOFA) score) at the time of diagnosis of sepsis (t0), and 48 hours (t48) and 120 hours (t120) later. Data in box plots are given as median, 25^th ^percentile, 75^th ^percentile and the 1.5 interquartile range. Outliers are shown in form of circles (1.5 to 3 interquartile ranges above 75^th ^percentile or below 25^th ^percentile) or rectangles (>3 interquartile ranges above 75^th ^percentile or below 25^th ^percentile).

**Figure 3:**
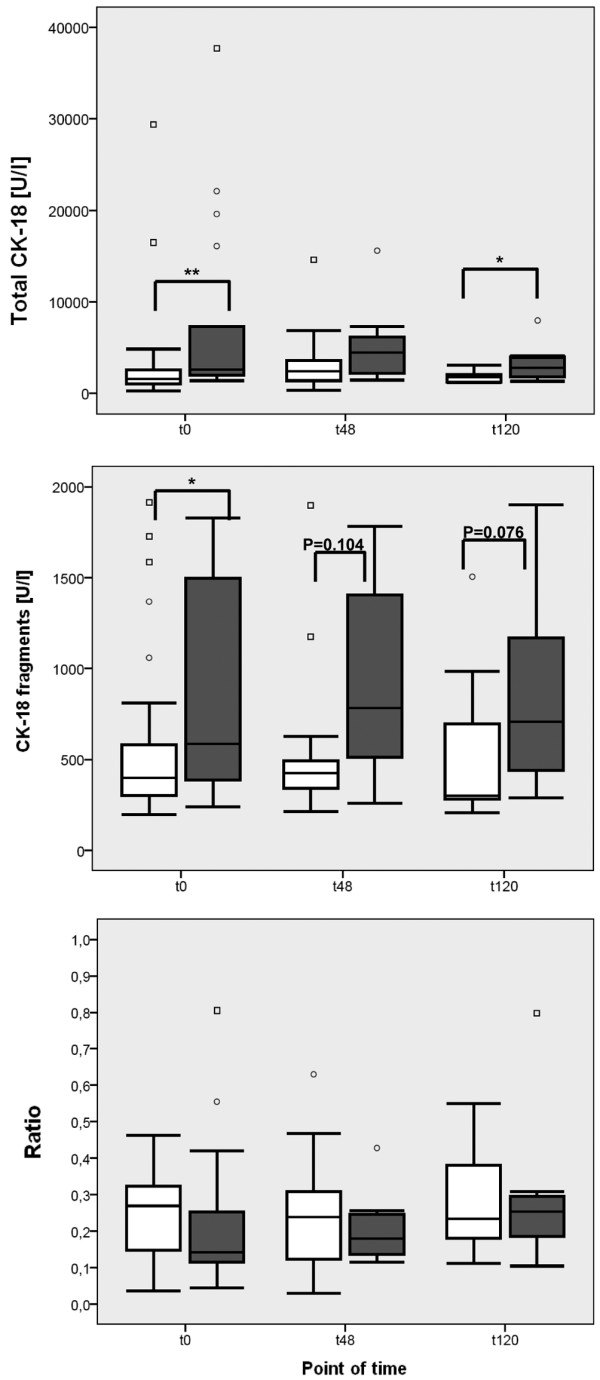
Comparison of cytokeratin measurements in patients with impaired and preserved liver function in the septic group at baseline and at 48 and 120 hours. Concentrations were measured of total cytokeratin-18 (CK-18) and CK-18 fragments (CK-18-F), and the ratio of CK-18-F to total CK-18 was calculated from the sera of patients with sepsis and impaired liver function ('Liver-Sequential Organ Failure Assessment (SOFA) ≥1', bilirubin ≥1.2 mg/dL or 20.5 μmol/L according to SOFA score, dark grey box) compared with patients with sepsis and preserved liver function ('Liver-SOFA = 0', bilirubin <1.2 mg/dL or 20.5 μmol/L according to SOFA score, white box) at the time of diagnosis of sepsis (t0), and 48 hours (t48) and 120 hours (t120) later. Data in box plots are given as median, 25^th ^percentile, 75^th ^percentile, and the 1.5 interquartile range. Outliers are shown in form of circles (1.5 to 3 interquartile ranges above 75^th ^percentile or below 25^th ^percentile) or rectangles (>3 interquartile ranges above 75^th ^percentile or below 25^th ^percentile). * *P *< 0.05: ** *P *< 0.01.

In patients with sepsis and preserved liver function (bilirubin < 1.2 mg/dL or 20.5 μmol/L, according to SOFA score), levels of total CK-18 and CK-18 fragments were comparable between the surviving and non-surviving subgroups at each time point (data not shown). Cytokeratin measurements in patients with sepsis who had no preexisting hepatic dysfunction (serum bilirubin <1.2 mg/dL or 20.5 μmol/L, according to SOFA score prior to the onset of sepsis syndrome) but sepsis-induced hepatic dysfunction are shown in Figure 2 by survivor and non-survivor status. Non-surviving patients with sepsis and impaired hepatic function showed a considerable trend toward increased levels of total CK-18 (Figure 2), whereby levels of CK-18 fragments remained comparable (Figure 2). When comparing cytokeratin measurements in patients with sepsis and either impaired (bilirubin ≥1.2 mg/dL or 20.5 μmol/L → liver-SOFA ≥1) or preserved (bilirubin < 1.2 mg/dL or 20.5 μmol/L → liver-SOFA = 0) liver function, Figure 3 shows significantly increased levels of total CK-18 and CK-18 fragments in septic patients with an impaired liver function, in comparison with patients with sepsis and preserved liver function. Furthermore, there was a high correlation (r = 0.72, according to Pearson's correlation analysis) in non-surviving patients with severe sepsis between the levels of total CK-18 and the levels of bilirubin 120 hours after the diagnosis of sepsis. In a comparable manner, levels of sICAM-1 were also highly correlated (r = 0.74, according to Pearson's correlation analysis) with the levels of bilirubin in the non-surviving subgroup, whereas levels of CK-18 fragments, IL-6, and sVCAM-1 failed to show such a correlation. In the surviving subgroup of patients with severe sepsis, there was no correlation between bilirubin and either CK-18 fragments or total CK-18 (data not shown).

In patients with severe sepsis and hepatic dysfunction, ROC curve revealed a cut-off value for total CK-18 at the onset of sepsis syndrome (Area under the curve = 0.78) of 1900 U/l for early discrimination of survivors and non-survivors with a sensitivity of 0.92 and a specificity of 0.60. In contrast, such a cut-off value could not be calculated for isolated CK-18 fragments.

In addition, serum levels of lactate and the amount of required vasoactive medication (norepinephrine) revealed a weak correlation (0.2<r < 0.5, according to Pearson's correlation analysis) with the levels of either total CK-18 or CK-18 fragments in the group of all severe septic patients, as well as in the different subgroups.

## Discussion

Severe sepsis, septic shock, and related multiple organ dysfunction syndrome is still the most common cause of death in intensive care medicine [1-6]. Many of the pathophysiologic changes during sepsis are related to inflammation [35]. Not surprisingly, different markers of systemic inflammation (eg, sICAM-1, sVCAM-1, IL-6) are significantly elevated during ongoing sepsis [34], whereas only IL-6 differed between patients after major abdominal surgery and healthy volunteers. This reflects generalized infection during sepsis, while patients after major abdominal surgery experience only mild activation of their inflammatory system [36].

As in many other diseases, it is still unclear whether necrosis or apoptosis plays the predominant role in severe sepsis. Although overlapping forms of the two modes of cell death can be observed [21], determination of the prevalent mode of cell death would be of great value because this may serve as an early prognostic marker for outcome in sepsis syndrome. Furthermore, newly developed therapeutic agents (eg, antiapoptotic drugs such as caspase-inhibitors) may represent potential therapeutic options in optimizing the unsatisfactory outcome of patients with severe sepsis [37].

The necrotic mode of cell death is independent of ATP, leads to uncontrolled release of cellular constituents, and is associated with a subsequent inflammatory response. Currently, detection of necrotic cell death involves measuring intracellular components (eg, in the liver, alanine and aspartate aminotransferases; in the heart, creatine kinase and troponin T), which are released into the serum during ongoing necrosis. These markers are widely used in clinical practice. However, the results of Bantel and colleagues [38] remind us that measuring different modes of cell death may provide additional information. In patients with chronic hepatitis C, levels of apoptotic cell death were increased, despite normal levels of serum aminotransferases, suggesting a non-critical stage of preexisting liver disease. Increased levels of apoptosis were associated with significant liver damage on liver biopsy. Therefore, it was concluded that the ensuing responses of cell repair, inflammation, regeneration, and fibrosis may all be triggered by apoptosis [39-42].

Apoptosis represents a strictly ATP-dependent, controlled form of cell death without inducing an inflammatory response [43,44]. As expected, this mode of cell death is more frequent in healthy people than in patients after trauma or surgery, or with sepsis. We observed a higher ratio of apoptosis to necrosis in healthy volunteers.

Key mediators of the apoptotic mode of cell death are caspases, intracellular proteases that cleave after aspartate residues. The resulting protein fragments represent new epitopes for which antibodies can be developed [25]. Caspases are activated via two different signaling routes [45,46]. The intrinsic pathway is related to the mitochondrial release of cytochrome C, whereas the extrinsic pathway is induced by death-mediating receptors [47,48]. Once caspases are activated, they cleave intracellular proteins, some of which are cell-type specific. CK-18, an intermediate filament protein, represents about 5% of the total protein content in most simple epithelial and parenchymal cells [22,23]. It is abundant in hepatocytes but is also present in the kidney, gut, colon, and lung. Apoptosis leads to early cleavage of CK-18 in position 238VEVD-A via the caspases 3, 6, and 7 and in position 396DALD-S mediated by the caspases 3, 7, and 9 [24,49]. The 396DALD-S cleavage generates a new epitope.

The CK-18 fragments can be detected by a newly developed M30 antibody. Increased levels of CK-18 fragments using this noninvasive parameter for evaluating apoptosis have already been described in patients with acute and chronic liver diseases [38,50-53], graft-versus-host disease [54], infectious gastroenteritis [52], and carcinoma [28]. Reports of increased apoptotic turnover in gastrointestinal epithelial cells of patients with sepsis [10,11] and a possible detrimental influence on survival [8] provided the first indication of an increased significance of apoptosis in the pathophysiology of sepsis. Sepsis was suspected of accelerating physiologic apoptotic turnover of cell types with a preexisting high rate of apoptosis, such as the gastrointestinal epithelium [55]. As a consequence of accelerated loss of gastrointestinal epithelial cells, the intestinal wall losses its barrier function with subsequent leakage of endotoxin and bacteria into the systemic circulation [56,57].

Increased levels of CK-18 fragments indicating elevated apoptotic turnover in critically ill patients was first described by Roth and colleagues [14]. Our observations agree with those results, which showed increased levels of CK-18 fragments in patients with sepsis compared with healthy volunteers and patients after trauma. In contrast, levels of CK-18 fragments in healthy volunteers and patients after trauma were comparable. These results can also be supported by our investigation, showing a comparable amount of CK-18 fragments in postoperative patients (ie, after surgical trauma) and healthy volunteers. Different traumatic effects (eg, surgical trauma versus bone fractures or organ rupture) seem to lead to increased necrotic cell death. The necrotic mode of cell death can be assessed indirectly by measuring cleaved CK-18 and uncleaved CK-18 (total CK-18) by using the M65 ELISA. In patients after surgical trauma we showed increased levels of total CK-18, and in agreement with Roth and colleagues no influence on apoptosis at early stages after trauma [14].

In addition to increased apoptotic turnover, patients with sepsis also showed significantly increased necrotic cell death as indirectly assessed by the M65 ELISA. In particular, non-survivors with impaired hepatic function showed a considerable trend toward higher levels of total CK-18. Furthermore, the levels of total CK-18 were highly correlated with the levels of bilirubin. Therefore, we assessed the ability of total CK-18 and CK-18 fragments to predict mortality in patients with severe sepsis, especially in those with an impaired hepatic function, because reliable prognostic parameters are still rare. Weigand and colleagues already described that ICAM-1 may exhibit the ability to predict mortality in septic shock, whereas endotoxin, IL-6, and other different circulating adhesion molecules (e.g. soluble L-selectin, soluble P-selectin, soluble E-selectin) failed to be of prognostic value [34]. Our investigation now demonstrates for the first time, that the measurement of caspase-cleaved and uncleaved CK-18 (total CK-18) appears to be an early predictor for survival in patients with severe sepsis and hepatic dysfunction.

Indeed, whether the presence of liver dysfunction is completely responsible for the observed differences of total CK-18/CK-18 fragments between survivors and non-survivors remains unclear. As described earlier, this might be due to the strict ATP-dependence of apoptosis, whereas necrosis also occurs without ATP [43]. In a mouse model of acetaminophen-induced hepatocellular dysfunction, Kon and colleagues demonstrated that necrosis was reduced when ATP depletion was prevented, whereas caspase-dependent apoptosis became more frequent [58]. Furthermore, ATP-depletion-induced necrosis as a result of mitochondrial dysfunction is consistent with high lactate levels in critically ill patients, especially those with acute liver failure, and is associated with a poor outcome [59].

Therefore, our observations are in agreement with the investigation by Volkmann and colleagues who showed that necrosis and not apoptosis seems to be the more frequent mode of cell death in critically ill patients with acute liver failure [53]. Furthermore, they showed an association between increased caspase activation and improved outcome in patients with acute liver failure. Therefore, it was concluded that caspases are not only key mediators of apoptotic cell death, but they also influence processes related to cell differentiation and proliferation. Because of these possible effects related to cell regeneration, the purpose of antiapoptotic drugs (eg, caspase-inhibitors) in critically ill patients has to be critically questioned, especially in the early phase of ongoing sepsis with increased apoptotic turnover [53]. All the more, it remains very important to accomplish optimal early goal-directed therapy, as promoted by Rivers and colleagues [30]. To optimize central venous pressure, mean arterial pressure, and central venous oxygen saturation, patients with sepsis must be treated aggressively with volume expansion (eg, crystalloids, colloids, and red blood cells) and with catecholaminergic regimens. The whole purpose of this strategy is to prevent the ATP-independent necrotic mode of cell death in critically ill patients with sepsis by achieving a balance between systemic oxygen delivery and oxygen demand.

## Conclusion

In summary, we have demonstrated that in healthy volunteers, cellular turnover occurred primarily by the apoptotic mode of cell death. Postoperative patients after major abdominal surgery showed comparable levels of apoptotic activity; in addition, the necrotic mode of cell death was markedly increased due to surgical tissue injury. In contrast, patients with severe sepsis showed increased levels of markers for both apoptotic and necrotic modes of cell death. The impact of these observations with regard to possible therapeutic options (such as caspase inhibitors) has to be critically questioned, because the loss of parenchymal cells due to necrosis may be the leading mode of cell death, especially in non-surviving patients with sepsis-induced hepatic dysfunction. In these patients, the measurement of caspase-cleaved and uncleaved CK-18 appears to be an early predictor for survival.

## Key messages

• In healthy volunteers, the mode of cellular turnover was primarily apoptotic cell death.

• Postoperative patients showed comparable levels of apoptotic activity, but necrotic cell death was markedly increased, probably due to surgical tissue injury.

• Patients with severe sepsis showed increased levels of markers for both apoptotic and necrotic cell death.

• In patients with severe sepsis and liver dysfunction, necrosis is increased relative to septic patients with intact hepatic function.

• The measurement of caspase-cleaved and uncleaved CK-18 appears to be an early predictor for survival in severe septic patients with hepatic dysfunction.

## Abbreviations

APACHE II: Acute Physiology and Chronic Health Evaluation II; CK-18: cytokeratin-18; ELISA: enzyme-linked immunosorbent assay; IL-6: interleukin-6; ROC: receiver operator curve; sVCAM-1: soluble vascular cell adhesion molecule-1; SOFA: Sequential Organ Failure Assessment; sICAM-1: soluble intercellular adhesion molecule-1.

## Competing interests

The authors declare that they have no competing interests.

## Authors' contributions

SH conceived of the study, participated in its design and coordination, and helped to draft the manuscript. TB performed data acquisition, carried out the ELISA measurements in the laboratory, drafted and wrote the manuscript, and prepared the tables and figures. CB participated in the design of the study and has been involved in revising the manuscript critically. JS performed data acquisition, participated in the design of the study, and was involved in revising the manuscript critically. CL participated in the design of the study and has been involved in revising the manuscript critically. JW participated in the design of the study and has been involved in revising the manuscript critically. TB participated in the design of the study, performed the statistical analysis, and was involved in revising the manuscript critically. EM participated in the design of the study and has been involved in revising the manuscript critically. UH conceived of the study, participated in its design, coordinated and helped to draft the manuscript. MW conceived of the study, participated in its design, coordinated and helped to draft the manuscript. Dr. Hoffmann and Dr. Weigand share senior authorship. All authors read and approved the final manuscript.
